# The first community outbreak of COVID-19 in Viet Nam: description and lessons learned

**DOI:** 10.5365/wpsar.2020.11.2.016

**Published:** 2019-04-27

**Authors:** Tran Nhu Duong, Le Thi Quynh Mai, Nguyen Tran Hien, Ngu Duy Nghia, Nguyen Trong Khoa, Nguyen Hai Tuan, Tran Anh Tu, Ngo Huy Tu, Hoang Vu Mai Phuong, Dang Duc Anh

**Affiliations:** a National Institute of Hygiene and Epidemiology, Ministry of Health, Hanoi, Viet Nam.; b Agency of Health Examination and Treatment, Ministry of Health, Hanoi, Viet Nam.

## Abstract

**Objective:**

At the time of this study, the prevention of novel coronavirus disease 2019 (COVID-19) relied solely on nonpharmaceutical interventions. Implementation of these interventions is not always optimal and, consequently, several cases were imported into non-epidemic areas and led to large community outbreaks. This report describes the characteristics of the first community outbreak of COVID-19 in Viet Nam and the intensive preventive measures taken in response.

**Methods:**

Cases were detected and tested for SARS-CoV-2 by real-time reverse transcriptase polymerase chain reaction. Contact tracing and active surveillance were conducted to identify suspected cases and individuals at risk. Clinical symptoms were recorded using a standardized questionnaire.

**Results:**

In Vinh Phuc province from 20 January to 3 March 2020, there were 11 confirmed cases among 158 suspected cases and 663 contacts. Nine of the confirmed cases (81.8%) had mild symptoms at the time of detection and two (18.2%) were asymptomatic; none required admission to an intensive care unit. Five prevention and control measures were implemented, including quarantining a community of 10 645 individuals for 20 days. The outbreak was successfully contained as of 13 February 2020.

**Discussion:**

In the absence of specific interventions, the intensive use of combined preventive measures can mitigate the spread of COVID-19. The lessons learned may be useful for other communities.

In December 2019, an outbreak of a novel coronavirus disease was reported from Wuhan, China, in association with cases of severe pneumonia, and originally thought to be connected to a seafood market. (*1*) Novel coronavirus disease 2019 (COVID-19), caused by the pathogen severe acute respiratory syndrome coronavirus 2 (SARS-CoV-2), has subsequently spread all over the world, with 1.5 million deaths as of early December 2020. (*2*, *3*) Viet Nam shares a 1200 km border with China, previously had multiple direct flights from Wuhan, and has had long-standing cultural and business ties with China, resulting in an increased risk of importation of SARS-CoV-2.

The first COVID-19 case in Viet Nam was detected in Ho Chi Minh City on 22 January 2020. The patient was a Chinese businessman from Wuhan visiting his son, who subsequently became infected. Shortly thereafter, a cluster of COVID-19 cases was detected among Vietnamese workers returning to the northern province of Vinh Phuc after 3 months of corporate training in Wuhan. In the absence of approved, effective vaccines or therapeutics, intensive preventive measures were the recommended response to cases of COVID-19. (*4*) This investigation describes the characteristics of the first community outbreak in Viet Nam and the intensive intervention and preventive measures taken in response.

## Methods

### Setting

Vinh Phuc province has an area of 1370.7 km^2^ and a population of 1 092 400 people. Binh Xuyen is one of seven districts in the province and includes 13 communes of approximately 10 000 people each. Vinh Phuc is approximately 51 km from Hanoi, the capital of Viet Nam, and home to 8 million people.

### Epidemiological investigation and laboratory methods

We defined cases of COVID-19 infection according to the Viet Nam Ministry of Health’s guidelines in effect at the time of our investigation. (*5*) Specifically, suspected cases of COVID-19 infection were people with fever and cough, with or without shortness of breath, and either (i) a history of visiting Wuhan, China, during the 14 days before onset of illness or (ii) close contact (within 2 m) with confirmed or suspected cases occurring from 17 January through 3 March 2020.

This investigation was conducted from 20 January to 3 March 2020. Confirmed cases were those who had laboratory confirmation of SARS-CoV-2 virus by real-time reverse-transcriptase polymerase chain reaction (rRT–PCR), (*6*) regardless of whether they had symptoms. Imported cases were defined as confirmed cases with a history of travel to an epidemic area within the 2 weeks before the date of onset of symptoms or the date of their first sample testing positive. Locally transmitted COVID-19 cases were defined as cases in Vinh Phuc province without a history of travel to an epidemic area. Symptoms were recorded at onset or time of first positive test result. The duration of hospitalization and clinical outcomes were monitored for all confirmed cases.

A close contact was defined as any individual who was within 2 m of a confirmed or suspected case during the case’s symptomatic period, including 3 days before symptom onset. A casual contact was defined as any individual who was further than 2 m from a confirmed or suspected case.

We conducted a descriptive epidemiological analysis by characterizing all cases in terms of their demographics, clinical symptoms, interval from onset to hospital admission, if applicable, number of contacts and history of travel to an epidemic area.

Oropharyngeal swabs were collected from suspected cases and all of their contacts, including those without symptoms. Testing by rRT–PCR was performed according to the Charité Institute of Virology’s protocol, as recommended by the World Health Organization. (*6*)

### Ethical considerations

This investigation was approved by the Institutional Review Board of the Pasteur Institute of Ho Chi Minh City, the organization with oversight of national research protocols for COVID-19.

## Results

The epidemiological characteristics were reported for 11 cases, 158 suspected cases and 214 close contacts. The intensive outbreak response, with its unique set of preventive measures, contributed to the successful containment of the COVID-19 outbreak.

### Epidemiology

The first community outbreak of COVID-19 occurred in Vinh Phuc province, where 11 cases were identified by contact tracing. To ensure complete case detection, attempts were made to identify all suspected cases between 30 January and 3 March 2020 – that is, from the day when the first case was detected to the last day of the lockdown.

#### Confirmed cases

**Figure 1:**
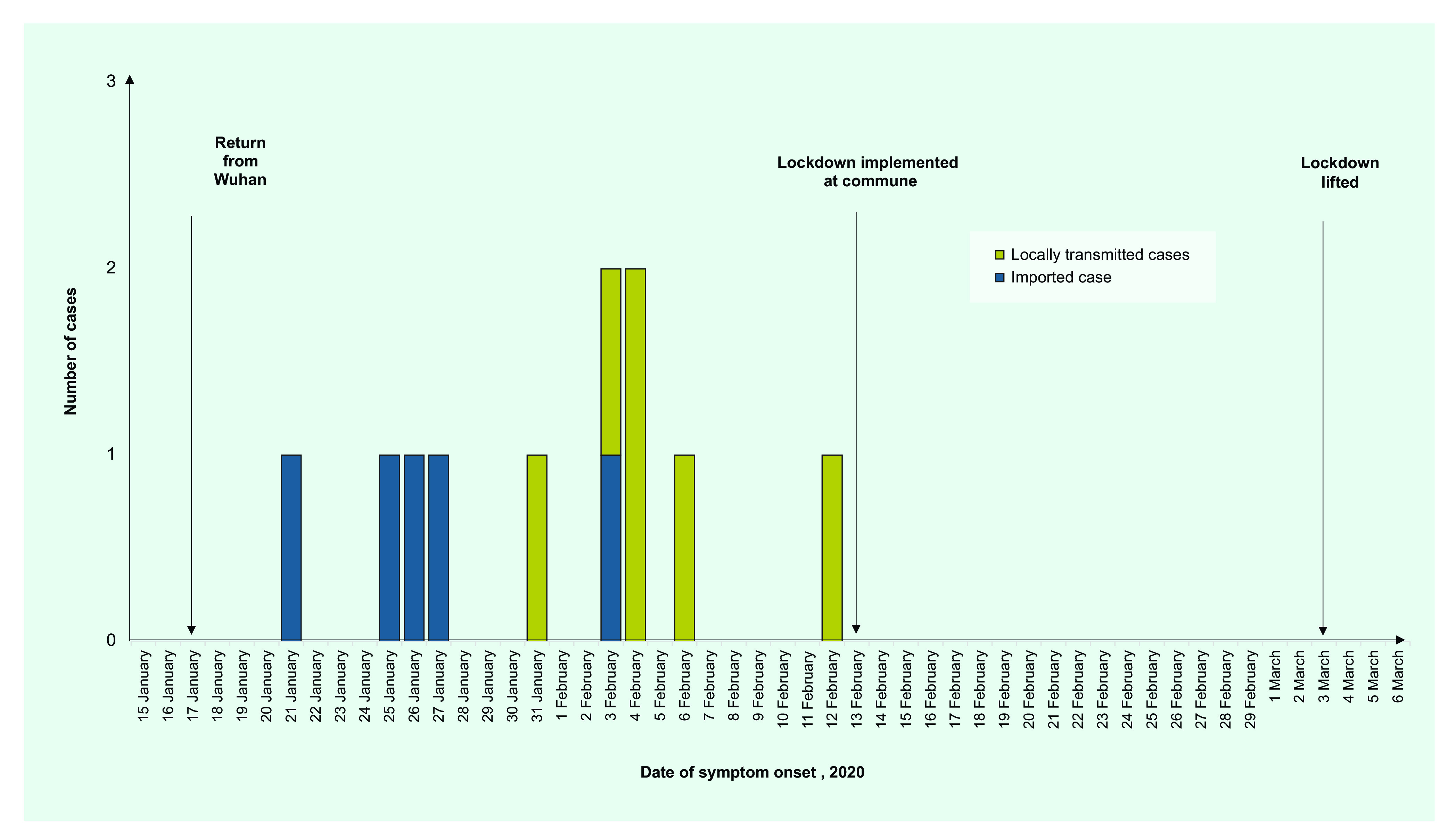
Epidemic curve of novel coronavirus 2019 (COVID-19) cases, by date of symptom onset, Vinh Phuc province, Viet Nam, January–February 2020

Of the 158 suspected cases of COVID-19, 11 cases were confirmed between 30 January and 3 March; the last confirmed case was identified on 12 February 2020 in Vinh Phuc province (**Table 1**). Five of these cases occurred among workers returning from Wuhan (imported cases) and the remaining six were close contacts (locally transmitted cases) of the imported cases (**Fig. 1**).

[**insert** Figure 1]

**Table 1:** Results of case finding and contact tracing for novel coronavirus disease (COVID-19), Vinh Phuc province, Viet Nam, January–March 2020

Location	No. of confirmed cases	No. of suspected cases with negative tests	No. of close contacts	No. of casual contacts
**All of Vinh Phuc province**	**11**	**147**	**214**	**449**
**Binh Xuyen district**	**9**	**99**	**149**	**200**
**Son Loi commune**	**6**	**40**	**70**	**52**
**All other communes**	**3**	**59**	**79**	**148**
**All other districts**	**2**	**48**	**65**	**249**

**Figure 2:**
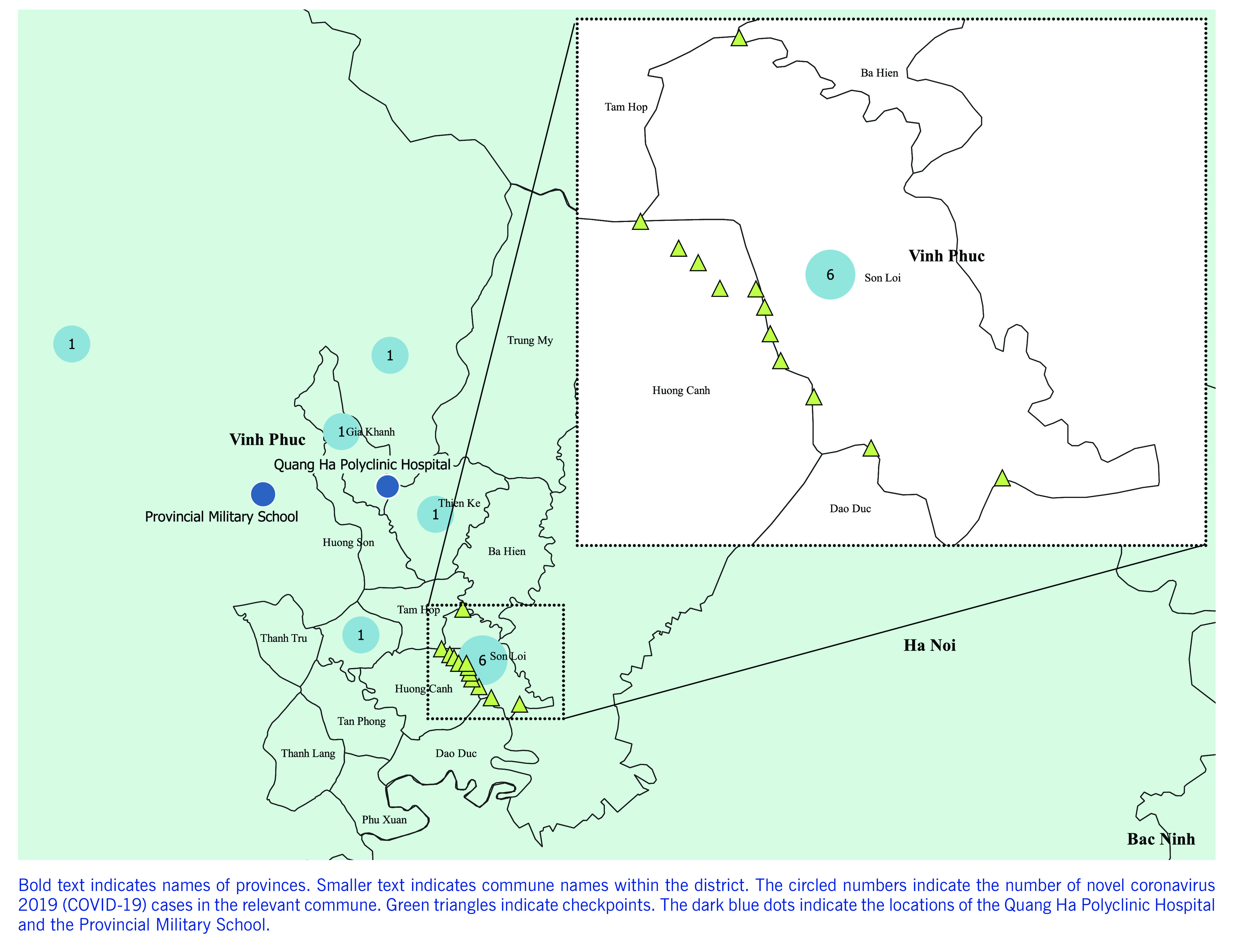
Map of Binh Xuyen district in Vinh Phuc province, Viet Nam, with an inset of Son Loi commune

Nine of the confirmed cases (81.8%) occurred among Binh Xuyen residents: three cases were imported and six were locally transmitted (**Table 1**). Two subsequent cases (cases 10 and 11) were identified through contact tracing and regular follow up. Of the two additional imported cases, one was a resident of the Tam Duong and one of the Tam Dao district (**Fig. 2**). Notably, all six locally transmitted cases could be linked either directly or indirectly to imported case number 2 (**Table 2**, **Fig. 1**). Of the 11 confirmed cases, 8 were female (72.7%) and 3 were male (27.3%); the median age was 29.0 years (interquartile range [IQR]: 26.5–45.5).

[**insert** Figure 2]

**Table 2:** Descriptive epidemiology of cases of novel coronavirus disease 2019 (COVID-19), Vinh Phuc province, Viet Nam, 17 January to 26 February 2020

Case	Gender	Age	Travel and contact history	Symptoms	Onset date	Hospital; date of admission	Date of discharge	Clinical outcome^b^
**1**	**Male**	**29 years**	**Travelled from Wuhan on 17 Jan**	**Cough**	**21 Jan**	**NHTD;** **23 Jan**	**18 Feb**	**Survived**
**2**	**Female**	**24 years**	**Travelled from Wuhan on 17 Jan**	**Fever, cough, sore throat**	**25 Jan**	**NHTD;** **26 Jan**	**10 Feb**	**Survived**
**3**	**Female**	**29 years**	**Travelled from Wuhan on 17 Jan**	**Fever**	**26 Jan**	**NHTD;** **2 Feb**	**10 Feb**	**Survived**
**4**	**Male**	**30 years**	**Travelled from Wuhan on 17 Jan**	**Fever, cough**	**27 Jan**	**NHTD;** **30 Jan**	**10 Feb**	**Survived**
**5**	**Female**	**42 years**	**Visited case 2's home on 22 and 28 Jan**	**Fever**	**31 Jan**	**QH Poly;** **31 Jan**	**18 Feb**	**Survived**
**6**	**Female**	**29 years**	**Travelled from Wuhan on 17 Jan**	**Asymptomatic**	**3 Jan**	**QH Poly;** **5 Feb**	**20 Feb**	**Survived**
**7**	**Female**	**49 years**	**Mother of case 2; same household**	**Cough**	**3 Feb**	**QH Poly;** **3 Feb**	**18 Feb**	**Survived**
**8**	**Female**	**16 years**	**Younger sister of case 2; same household**	**Asymptomatic**	**4 Feb**	**QH Poly;^a^** **5 Feb**	**20 Feb**	**Survived**
**9**	**Female**	**55 years**	**Visited case 2's home on 28 Jan**	**Fever, headache**	**4 Feb**	**QH Poly;^a^** **NHTD,** **5 Feb**	**18 Feb**	**Survived**
**10**	**Female**	**3 months**	**Stayed with case 2's family on 28–31 Jan**	**Cough, runny nose**	**6 Feb**	**QH Poly;^a^** **NPH,** **6 Feb**	**20 Feb**	**Survived**
**11**	**Male**	**50 years**	**Father of case 2; same household**	**Fatigue**	**12 Feb**	**QH Poly;** **11 Feb**	**26 Feb**	**Survived**

NHTD: National Hospital for Tropical Diseases; QH Poly: Quang Ha Polyclinic Hospital; NPH: National Paediatric Hospital.

^a^ These patients were first admitted to Quang Ha Polyclinic Hospital (QH Poly) then transferred to a national referral hospital to prevent complications; transfers included a patient with thrombocytopenia and a 3-month-old infant.

^b^ The outcome “survived” refers to clinical outcome at the time of hospital discharge.

Nine confirmed cases (81.8%) had mild symptoms at the time of detection; no cases required admission to an intensive care unit. Four out of five cases with imported COVID-19 who had travelled to Wuhan were symptomatic. The first case developed symptoms on 21 January 2020, 4 days after returning from Wuhan (**Table 2**). The most common symptoms were cough or fever, found in 8/11 cases (72.7%). Two patients (18.2%) had both cough and fever (**Table 2**). The less frequent symptoms of sore throat, headache, runny nose and fatigue were each found in one patient (9.1% for each of the four symptoms). Two other confirmed cases (18.2%) were asymptomatic at the collection date of their first specimen for testing by rRT–PCR, but the specimen tested positive.

Through case finding, we observed a decrease in the number of days that cases spent in the community before being hospitalized, with a median of 2 days (IQR: 2–3) for the five imported cases (cases 1, 2, 3, 4, 6) and 0 days (IQR: 0–0.75) for the six locally transmitted cases (cases 5, 7, 8, 9, 10, 11). The difference in delay in hospital admission between imported cases and locally transmitted cases was statistically significant (Wilcoxon rank sum test: *P* = 0.011). The number of close contacts was not significantly different between the two groups (imported cases versus locally transmitted), with a median of 4 contacts (IQR: 4–6) of imported cases and 4.5 contacts (IQR: 4–5.75) of locally transmitted cases (*P* = 0.92). All cases recovered clinically, as assessed by the Vietnamese Ministry of Health’s guidelines, (*7*) and were discharged following two negative rRT–PCR tests of upper respiratory specimens collected at least 24 hours apart (**Table 2**).

#### Close contacts

A total of 214 close contacts were identified (**Table 1**). Six of these subsequently became confirmed cases, five of whom were tested and identified at home, and one, the father of case 2, who developed a sore throat and fatigue while quarantined at the local Provincial Military School (described below), who was immediately transferred to the Quang Ha Polyclinic Hospital where a specimen was collected and subsequently tested positive by rRT–PCR.

All 39 close contacts of the five imported cases were asymptomatic and were quarantined at the Quang Ha Polyclinic and the military school, as were 95 other close contacts without symptoms. The remaining 80 close contacts were quarantined at home.

### Outbreak response

The field outbreak response was led by the Vinh Phuc Provincial Centre for Disease Control with support from the National Institute of Hygiene and Epidemiology. The National Steering Committee for COVID-19 Prevention and Control also deployed an expert technical outbreak surveillance team, a rapid response team, an expert treatment team and an infection control team to Vinh Phuc province to support local authorities in directing, monitoring and implementing all prevention activities. Based on the descriptive epidemiology, interventions using a series of preventive measures were implemented.

Five doctors were deployed to each of the 13 commune health stations (CHSs) in the Binh Xuyen district (65 doctors in total) to ensure compliance with preventive measures. An additional 168 health-care workers at the district and commune levels were trained in case investigation, reporting, contact tracing, surface disinfection and the proper use of personal protective equipment (PPE).

#### Contact tracing

Contact tracing was performed by the Provincial Centre for Disease Control. All suspected cases were interviewed to collect information about their close contacts, including health-care contacts, family members, co-workers, friends, neighbours, other social contacts and travelling companions. All contacts were subjected to quarantine and strict symptom monitoring.

#### Isolation and quarantine

The five imported cases (cases 1–4, 6) were isolated and treated at the National Hospital for Tropical Diseases in Hanoi (**Table 2**), since they were among the first imported cases in Viet Nam.

The initial hospital isolation and treatment implemented in Vinh Phuc province occurred at the Quang Ha Polyclinic Hospital, a district hospital in Binh Xuyen. It was divided into six sections, one each for:

isolation and treatment of laboratory confirmed cases;
suspected cases with pending test results;
family members of confirmed cases;
symptomatic patients whose first COVID-19 test was negative but who required 14 days of observation;
those who had recovered fully from COVID-19; and
suspected cases and close contacts who tested positive for influenza or other respiratory viruses.

Patients in the isolation facility had their temperature and symptoms checked twice daily. For those with symptoms, temperature and symptom checks were performed four times per day. Suspected cases from other districts were isolated at the Vinh Phuc Provincial Hospital.

The local Provincial Military School was converted into a quarantine centre for close contacts who were not family members of cases. Beds were placed 1 to 2 m apart. Those who were quarantined or isolated received three meals a day free of charge and full support and daily supplies. Waste was separated into potentially contaminated waste (e.g. masks and tissues) and all other waste. Temperature and symptom checks were conducted twice daily. We collected oropharyngeal specimens for laboratory testing from each contact under quarantine, once on day 2 and once on day 14 before discharge. We delivered risk communication messages to all quarantined contacts each day.

Four suspected cases were identified in the facility and were transferred to Quang Ha Polyclinic. One of these four suspected cases became case 11. All discharged contacts from the quarantine centre remained under home quarantine for 2 more weeks.

No locally transmitted cases were identified among health-care workers in the Quang Ha Polyclinic or among staff at the military school quarantine centre.

#### Community lockdown

Intensive lockdown measures were taken after the identification of three locally transmitted cases in the Son Loi commune on 7 February. We worked with local authorities and implemented preventive control measures in the commune. A 20-day lockdown of the entire commune of 10 645 residents began with the establishment of eight checkpoints on 8 February and four more were added between 9 and 13 February. The lockdown officially started at midnight on February 13.

Twelve checkpoints were established by 14 February and were in place until 3 March (**Fig. 2**). The checkpoints were inspected regularly by 30 independent monitoring teams designated by the Provincial Steering Committee for COVID-19 Prevention and Control. Residents of Son Loi were permitted to leave for work in nearby fields or emergency purposes, but they were required to register at checkpoints and inform local authorities of when they would return. Visitors were only permitted to deliver supplies (e.g. food, water) to the checkpoints, from which they were collected and distributed within the commune. All task force staff and visitors without symptoms and with a forehead temperature < 37.5 °C were permitted to enter.

Merchandise and vehicles entering and exiting Son Loi were inspected and disinfected with 0.1% chloramine B solution. Shops with fixed prices for staple foods, such as rice, noodles, meat and vegetables, were established during the lockdown in each of the six hamlets of Son Loi.

Each member of the commune received a daily allowance of 40 000 Vietnamese dong (US$ 1.70) for the 20-day duration of the lockdown. Residents were recommended to clean their houses and domestic surfaces daily with 0.1% chloramine B solution, wear masks and stay home as much as possible. Mass gatherings, such as festivals and weddings, were prohibited during the lockdown. Risk communication messages were delivered three times a day via loudspeakers throughout the commune.

A team of medical experts was sent to the Son Loi CHS to support the rapid identification of suspected cases and to meet any emergency needs of the residents. Two ambulances were always on duty at the CHS. A mobile X-ray unit was acquired by the Son Loi CHS, a device not available at most CHSs in Viet Nam.

#### Active case finding

Active case finding was performed during the lockdown. A total of 29 Community COVID-19 Prevention and Control Teams (CPCTs) were formally established. The teams consisted of three or four members, and included village health-care workers, volunteers and community or family representatives. The teams performed daily house-to-house health checks, including taking the temperature of all household members and delivering risk mitigation messages. Each household was provided with a thermometer so that symptomatic family members could have their temperature assessed and reported to the team by calling a dedicated phone number. No cases were identified during active case finding.

#### General preventive measures

For people in the commune, general preventive measures were required at all times, including wearing masks, using other PPE, disinfecting surfaces and using hand sanitizer. In addition to the recommended general preventive measures, all hospitalized patients, quarantined individuals, suspected cases and close contacts of confirmed cases were also required to wear masks at all times.

All staff working at the CHSs, the military school and Quang Ha Polyclinic; members of the CPCTs; and personnel at other medical facilities in Vinh Phuc consistently wore a complete set of PPE, including a whole-body suit, gloves, eye protection and a surgical mask. All were encouraged to practice hand hygiene regularly, before and after meals, before and after caring for patients, and after using the toilet. Surface disinfection of hallways with 0.1% chloramine B solution was performed daily in health-care and quarantine facilities. All vehicles entering and exiting the military school campus, cars transporting suspected cases and ambulances were disinfected daily with 0.1% chloramine B solution.

## Discussion

During the first community outbreak of COVID-19 in Viet Nam, 11 of 158 (6.9%) suspected cases tested positive for COVID-19, indicating a low rate of infection. (*8*) These 11 cases were identified in people who returned from Wuhan or were in close contact with one of these people. Given the transmissibility of SARS-CoV-2, this cluster had the potential to be much larger. (*4*, *5*)

The majority of clinical manifestations in the confirmed cases included cough or fever, or both. (*9*) About 20% of cases were asymptomatic, a low prevalence compared with previous reports. (*10*) The time from onset of symptoms or detection of a case to hospital admission for isolation and treatment was short, most likely due to the careful monitoring of suspected cases and close contacts at the hospital and at the quarantine centre. Since cases may be infectious for 1–3 days before symptom onset and thus contribute to community transmission, (*11*) it is crucial to identify both symptomatic and asymptomatic cases for isolation and quarantine. (*12*-*14*) The interval from symptom onset to hospital admission or isolation was reduced from 2 days to 0 during this outbreak as a result of efforts by local public health staff to limit the spread of cases. The delay between symptom onset and isolation has been shown to have the largest role in determining the degree of community transmission from imported cases. (*15*) Therefore, early detection and careful monitoring of suspected cases and close contacts can reduce the time that potential cases spend in the community; by committing to early detection and careful monitoring, Vinh Phuc province may have limited the spread of COVID-19 during the first community outbreak in Viet Nam.

The control measures implemented in response to this outbreak occurred 3 days after locally transmitted cases were identified in the community. This quick response was feasible with the government’s assistance and because of the informed decisions made in near real-time by the National Steering Committee as its rapid response team was deployed to Vinh Phuc province. The establishment of quarantine and treatment facilities at the district level facilitated and supported timely case detection, contact tracing and quarantining of people at risk, which may have contributed to reducing the spread of COVID-19 in the community. The decision to implement a community lockdown for 20 days was supported by Vietnamese government Decree No. 101/2010/ND-CP. (*16*) Similar measures were implemented in China in 2003 in response to severe acute respiratory syndrome (*17*) and, more recently, in response to COVID-19 in Singapore. (*11*) Recent analysis suggests that increased compliance with community mitigation strategies, including physical distancing, when the number of cases is increasing can reduce community transmission. (*18*) When first implemented, large-scale lockdown of communities can be highly disruptive. However, if it is implemented quickly and at a smaller scale, that disruption can be minimized and, importantly, disease transmission is more likely to be contained. In Son Loi commune, the lockdown was implemented for no longer than necessary, and the reason for it was to reduce pressure on the health, economic and social security of the people in the commune. (*19*)

Our investigation took place at a time when there was no effective vaccine or treatment and the national public health response in Viet Nam was still developing. This created several challenges. For example, at the time when the employees returned from Wuhan, there was no national or international guidance on how to detect or manage asymptomatic cases. Therefore, we had to adopt what we believed to be sensible public health interventions, assuming that asymptomatic cases could transmit SARS-CoV-2 and, thus, isolating them as if they were infectious. The conventional rRT–PCR assay was not readily available at central laboratories before 30 January 2020, so we often erred on the side of isolation and quarantine, knowing that suspected cases and contacts might have to wait several days for test results.

Our investigation began near the time of the annual Lunar New Year (Tet) holiday, the largest holiday in Viet Nam and a time when most government offices and businesses are closed. As such, we were not able to access all available resources. Nevertheless, despite these challenges, we were able to contain the outbreak in Vinh Phuc and prevent further transmission. The experiences gained through the response to this outbreak were indispensable for the development of subsequent national guidelines. (*20*-*23*)

This investigation has several limitations. The five cases with a history of travel to an epidemic area were considered imported cases; however, we were unable to determine when and where they were infected with the SARS-CoV-2 virus. The transmission of pathogens among these five cases was unclear. Furthermore, the effectiveness of each preventive measure was not separately assessed, so we do not know exactly which measures played key roles in the combined intervention.

In conclusion, in COVID-19 response activities, the government’s assistance and the willingness of the community to adopt preventive measures are important in containing community outbreaks. When no vaccine is available, intensive interventions that involve a combination of preventive measures can mitigate spread of the disease. We believe that these experiences are useful for other communities that may need to respond to the COVID-19 pandemic.
